# Assessment of Cell Viability in Drug Therapy: IC50 and Other New Time-Independent Indices for Evaluating Chemotherapy Efficacy

**DOI:** 10.3390/pharmaceutics17020247

**Published:** 2025-02-13

**Authors:** Marta Sánchez-Díez, Paula Romero-Jiménez, Nicolás Alegría-Aravena, Clara E. Gavira-O’Neill, Elena Vicente-García, Josefa Quiroz-Troncoso, Raquel González-Martos, Carmen Ramírez-Castillejo, Juan Manuel Pastor

**Affiliations:** 1 CTB (CTB-UPM) Centro de Tecnología Biomédica, Universidad Politécnica de Madrid, 28223 Pozuelo de Alarcón, Spain; paula.romero.jimenez@alumnos.upm.es (P.R.-J.); clara.gavira@ctb.upm.es (C.E.G.-O.); elena.vicente.garcia@alumnos.upm.es (E.V.-G.); josefa.quiroz@ctb.upm.es (J.Q.-T.); raquel.gonzalez@ctb.upm.es (R.G.-M.); 2 Grupo de Sistemas Complejos, Universidad Politécnica de Madrid, 28040 Madrid, Spain; juanmanuel.pastor@upm.es; 3 Instituto de Desarrollo Regional (IDR) and Instituto de Investigación en Recursos Cinegéticos (IREC), Universidad de Castilla-La Mancha (UCLM), 02071 Albacete, Spain; nicolas.alegria@uclm.es; 4 Asociación Española Contra el Cáncer (AECC)-Fundación Científica AECC, 02004 Albacete, Spain; 5 Nageru S.L., 28045 Madrid, Spain; 6 Escuela Técnica Superior de Ingeniería Agronómica, Alimentaria y de Biosistemas, Departamento Biotecnología-Biología Vegetal, Universidad Politécnica de Madrid, 28040 Madrid, Spain; 7 Departamento de Oncología, Instituto de Investigación Sanitaria San Carlos (IdISSC), 28040 Madrid, Spain; 8 Grupo Interdisciplinar de Sistemas Complejos (GISC), 28040 Madrid, Spain

**Keywords:** effective growth rate, mathematical model, cell viability, IC50, drug resistance

## Abstract

**Background/Objectives**: Cell viability assays play a crucial role in cancer research and the development of effective treatments. Evaluating the efficacy of conventional treatments across different tumor profiles is essential for understanding patient resistance to chemotherapy and relapse. The IC50 index has been commonly used as a guide in these assays. The idea behind the IC50 index is to compare cell proliferation under treatment with respect to a control population exposed to the same treatment. The index requires normalization to a control and is time dependent. These aspects are disadvantages, as small variations yield different results. In this article, we propose a new method to analyze cell viability assays. **Methods**: This method involves calculating the effective growth rate for both control (untreated) cells and cells exposed to a range of drug doses for short times, during which exponential proliferation can be assumed. The concentration dependence of the effective growth rate gives a real estimate of the treatment on cell proliferation. A curve fit of the effective growth rate related to concentration yields the concentration corresponding to a given effective growth rate. **Results**: We use this estimation to calculate the IC50 index and introduce two new parameters (ICr0 and ICrmed) to compare treatment efficacy under different culture conditions or cell lines. **Conclusions**: In summary, this study presents a new method to analyze cell viability assays and introduces two more precise parameters, improving the comparison and evaluation of efficacy under different conditions.

## 1. Introduction

Cancer is one of the most widely studied diseases in contemporary medical research. The collective burden of cancer is substantial, encompassing both prevalence and consequential socio-economic impact. The World Health Organization (WHO) estimated it to be one of the leading causes of death worldwide, reaching 35 million new cases by 2050. Cancer can impact individuals across all age groups and is associated with a wide range of risk factors, including genetics, exposure to carcinogenic substances, lifestyle, and other environmental factors [1]. Its multifaceted nature and pervasive impact on global health have triggered an enduring search for deeper insights into its mechanisms and potential therapeutic interventions.

Globally, lung cancer has the highest incidence, followed by breast cancer and colorectal cancer. In terms of mortality, colorectal cancer is the second leading cause and breast cancer is the fifth most common cause of cancer death [2,3]. Despite the high incidence, conventional treatments have demonstrated high efficacy, leading to high 5-year survival rates in developed countries (65% in colorectal cancer and 90% in breast cancer, per the World Health Organization (WHO)) [1]. However, the prognosis depends on the stage of the disease, with the possibility of metastasis remaining an Achilles heel for treatment. Cancer progression is mainly driven by the occurrence of therapy resistance [4,5]. For this reason, there is a fundamental interest in understanding the efficacy of conventional treatments on different tumoral profiles.

To this end, cell viability assays have become instrumental tools for assessing the effectiveness of anti-cancer agents, particularly in the early stages of treatment studies. Diverse methodologies have emerged, driven by the need for a more comprehensive understanding of cellular responses. Traditionally focused on survival or death, these studies have expanded to analyze specific aspects of cellular function. Advanced methods, including thiazolyl blue tetrazolium bromide (MTT) assays, fluorescence microscopy, real-time cell analysis (RTCA), and flow cytometry, offer detailed assessments, particularly relevant in studying tumor cells and therapeutic compound efficacy [6]. The MTT assay, a widely used technique, is a cost-efficient method. It relies on the reduction in MTT to formazan, providing a colorimetric measure of cell viability by absorbance [7]. The fact that cell lines grow exponentially has been widely studied since 1961 [8,9,10] and continues to be used in recent studies [11,12]. In these experiments, the absorbance is usually normalized to the control cell line, so the evaluation of exponential growth in controls is essential to determine that there are no external factors that affect the culture. The percentage of viability is usually calculated as follows:

(1) $$\mathrm{Cell}\;\mathrm{viability}\;(\%)=\frac{\mathrm{Population}\;\mathrm{sample}}{\mathrm{Population}\;\mathrm{control}}x\;100=\frac{\mathrm{Absorbance}\;\mathrm{sample}}{\mathrm{Absorbance}\;\mathrm{control}}x\;100,$$

Among these assays, the discovery of parameters to analyze and compare the effectiveness of treatments is essential. The determination of the half-maximal inhibitory concentration (IC50) has gained prominence at this point. The IC50 value denotes the concentration of a compound at which 50% of cell viability is inhibited, serving as a parameter to assess the effectiveness of potential therapeutic compounds [13]. Its use in cell viability assays offers a quantitative measure, enabling researchers to compare the efficacy of different compounds and make informed decisions in the development of cancer treatments [14,15]. Determining IC50 from dose–response curves is essential but challenging. Various methods, including non-linear regression and statistical models, navigate curve complexities [13,16,17]. However, a major drawback of the IC50 index is its time-dependent nature. Since both the sample and control cell populations evolve over time at different growth rates, performing the same assay with different endpoints can result in different IC50 values.

**Figure 1:**
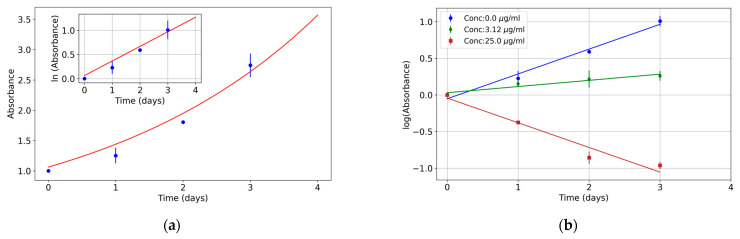
Exponential growth in the HCT116 cell line in control and treatment conditions. (**a**) MTT absorbance reading over time fits with an exponential curve in control conditions. The inset shows this growth on a semilogarithmic scale. (**b**) Exponential fitting of MTT absorbance readings on a semilogarithmic scale for different oxaliplatin concentrations (0, 3.32, and 25 µg/mL). Note that in a semilogarithmic scale plot, data points can be fitted with a linear regression. Three independent experiments were performed, each including three technical replicates.

In optimal laboratory conditions, cell cultures produce colonies with exponentially growing populations, with a well-defined growth rate [18,19]. When these conditions are changed, for example, to study chemotoxicity, some cells die and others reproduce at slower rates; however, the change in colony population growth dynamics can be modeled as proportional to the colony population itself (because it is a multiplicative phenomenon). The proportional constant is a new effective growth rate, i.e., the growth rate that can be measured for a population under given conditions. A plot of the colony population (or any proportional magnitude, such as MTT absorption) versus time shows exponential growth (in a log-scale plot, data points can be fitted with a linear regression) whose effective growth rate depends on culture conditions. Figure 1a shows a typical plot of an MTT assay with an exponential fit in the HCT116 human colorectal cancer cell line; the inset depicts the same data in a semi-logarithmic scale so that the exponential growth fit is shown as a linear fit. This is due to the well-known Malthus law. If the number of individuals in the population increases, the growth rate is positive, but if the number of living individuals decreases, it corresponds to a negative growth rate (exponential with negative exponent). Thus, a direct way to evaluate the effect of a chemotoxicant on a cell culture is to study the change in its growth rate. As an example, Figure 1b shows three exponential fits for different oxaliplatin concentrations in the same cell line; the growth rate (the slope of the fit) decreases as the concentration increases, and, finally, the slope becomes negative at high concentrations.

In this article, we propose a new method based on the calculation of the effective growth rate for a range of drug concentrations. Cell proliferation, at short times, can be modeled as an exponential function, whose characteristic parameter is the exponent, called growth rate. Thus, the cell population as a function of time and growth rate was defined as follows:

(2) $$N(t)=N_{0}\cdot e^{r\cdot t},$$

where r is the growth rate and N_0_ is the cell population at t = 0.

Since the colony population is an exponential function of time, the rate of two populations will similarly follow an exponential pattern. Consequently, the IC50 index will also vary with time. However, over short timeframes, the growth rate is a time-independent parameter with real biological meaning. Moreover, as the effective growth rate is dependent on drug concentration, we can estimate the IC50. We also propose the use of two new parameters to evaluate the response of cell lines to different treatments that are not time dependent. One of them is the ICr0 index, a value that represents the drug concentration at which the effective growth rate is zero. The other, the ICrmed, is the value that corresponds to the drug concentration that reduces the control population’s growth rate by half. Advancements in growth rate analysis offer a precise alternative for IC50 determination without succumbing to curve intricacies.

In summary, our study intertwines diverse threads of research, amplifying the interpretation of dose–response curves. By embracing innovative methods for IC50 determination and growth rate analysis, we aim to uncover the comprehensive narrative of treatment effects on cancer cells, optimizing therapeutic strategies in the multifaceted landscape of cancer treatment development.

## 2. Materials and Methods

### 2.1. Cancer Cell Lines and Cell Culture

Five human colorectal cancer cell lines and a human breast cancer cell line were employed: SW-480 (CCL-228), SW-620 (CCL-227), DLD-1 (CCL-221), HCT116 (CCL-247), HT29 (HTB-38), and MCF7 (HTB-22). All of them were purchased from the American Type Culture Collection (ATCC, Manassas, VA, USA). The cells were cultured in Dulbecco’s Modified Eagle Medium (DMEM, Corning, New York, NY, USA, Ref: 10-017-CV) supplemented with 10% fetal bovine serum (FBS, PAN Biotech, Aidenbach, Germany, Ref: P30-3302), 1% L-glutamine (PAN Biotech, Aidenbach, Germany, Ref: P04-82050), and 1% penicillin/streptomycin (Corning, New York, NY, USA, Ref: 30-001-Cl) at a 37 °C humidified incubator (Series II Water Jacket, Thermo Scientific, Waltham, MA, USA) with 5% CO_2_.

### 2.2. Cell Viability Assay

Thiazolyl blue tetrazolium bromide (MTT, BioChem, PanreacApplichem, Barcelona, Spain, Ref: A2231) assays were performed to detect the viability to different concentrations of oxaliplatin (50 μg/mL, 10 serial 1:2 dilutions starting from the maximum doses) or cisplatin (200 μM, 9 serial 2:3 dilutions starting from the maximum doses). Both chemotherapy reagents were purchased from Accord Healthcare S.L.U., Barcelona, Spain. Briefly, cells were seeded in 96-well plates (Deltalab S.L, Barcelona, Spain, Ref: D200005) at a concentration of 100,000 cells/mL in a volume of 100 μL. Next, the chosen chemotherapeutic drug was added to each well. Three replicates per condition were performed in each plate with three independent experiments in total. After 0 (seeding time), 24, 48, and 72 h of the treatment, the medium was removed, and 50 μL of 0.5 mg/mL MTT was added. Then, plates were incubated for 4 h at 37 °C, and finally, the medium was removed and resuspended in 100 μL of dimethyl sulfoxide (DMSO, Labkem, Barcelona, Spain, Ref: DMSO-0GH-2K5) before detection of absorbance in a BIOBASE-EL 10A (Biobase, Shandong, China) spectrophotometer at 546 nm. Three independent experiments were conducted for each cell line, containing a minimum of 3 technical replicates.

### 2.3. Mathematical Model

As shown in Figure 1, colony population can be fitted as an exponential growth for short times, with an effective growth rate that can be positive or negative (indicating a population that increases or declines over time, respectively). In cell viability experiments, the population of a colony subjected to a given concentration of chemotherapy drug, N_c_, will also follow an initial exponential growth, whose effective growth rate could be positive or negative. Then, cell viability can also be written as an exponential growth with an exponent equal to the difference between the sample growth rate and the control growth rate as follows:

(3) $$V(t)=\frac{N_{C}(t)}{N^{*}(t)}=\frac{N_{0C}\cdot e^{r_{C}t}}{N_{0}^{*}\cdot e^{r_{0}t}}=\frac{N_{0C}}{N_{0}^{*}}\cdot e^{\left(r_{C}-r_{0}\right)\cdot t},$$

where N_C_ and N_0_* are the respective initial populations of the colony under treatment and the control and r_c_ and r_0_ are the effective growth rates of the colony under treatment and the control, respectively. In this expression, one can see the time dependence of the viability. The greater the difference, the faster the viability will change over time. In any case, from this equation, the sample growth rate can be calculated for any viability value as follows:

(4) $$r_{C}=r_{0}+\frac{1}{t}\cdot ln\left(V(t)\cdot \frac{N_{0C}}{N_{0}^{*}}\right),$$

### 2.4. Growth Rate Calculation

The absorbances measured from controls and treated cells were used to calculate the growth rates in each condition. First, the data were normalized to the absorbance obtained in the seeding time (t0). Then, the absorbances from 24 h (t1), 48 h (t2), and 72 h (t3) were plotted, and the effective growth rates were obtained from linear fits by the least squares method. To estimate the confidence intervals of the effective growth rate, bootstrap methods with 1000 bootstrap samples were used [20].

Bootstrapping is a technique to estimate the properties of a population statistic from the properties of sampling data from an approximating distribution. The bootstrap idea is to use the sample distribution instead of the population distribution. The sample distribution is made empirically by resampling from the original data. For each bootstrap sampling, the same number of data is randomly selected with replacement from the original sample. With each bootstrap sample, the statistic of interest is calculated and finally, a distribution of the statistic is obtained. When data distribution is known or can be estimated, bootstrap sampling can be drawn from this distribution. In this case, the bootstrap method is called parametric. In regression problems, with a covariate and a response variable, observed data are formed by pairs (X_i, Y_i); in this case, the empirical bootstrap method (also called nonparametric bootstrap) consists of random sampling with replacement of the pairs from the observed data (in the regression model, empirical bootstrap is also called paired bootstrap). In an empirical bootstrap, covariate data values are independently randomly selected, so if sampling of all the predictors as fixed values is needed, one may use the residual bootstrap model. In this instance, one fits the observed data and determines the residuals for each observation. With the residual bootstrap technique, bootstrap samples are selected from the residuals, and the new $y_{i}^{*}$ is determined by $y_{i}^{*}=\hat{y_{i}}+e_{b}$, where $\hat{y_{i}}$ is the fitted value for x_i_ and e_b_ is an independently randomly selected bootstrap residual [21].

The effective growth rate is obtained from the slope distribution of all linear fits of the logarithm of MTT values versus time (one fit for each bootstrap sample) from a total of 1000 bootstrap samples. This slope distribution can be fitted to a Gaussian distribution, as the repetition of multiple iterations, as performed by the bootstrap method, is known to yield this type of distribution [20]. From the bootstrap distribution, one can obtain the effective growth rate (its mean, $\bar{r}$) and its standard error (SE). After verifying that the statistic is roughly normally distributed, a 100(1 − α)% confidence interval can be estimated as follows:

(5) $$r=\bar{r}\pm z_{\alpha /2}SE,$$

where $z_{\alpha /2}$ is the standard normal value with probability *α*/2 to the right.

Curve fittings simulations were carried out in Python (Python Software Foundation, Wilmington, DE, USA) with the *scipy* [22] and *pandas* [23] libraries. Graphics were carried out with the matplotlib library [24]. The Python scripts are available on GitHub (https://github.com/JJ-Lab/CSC_IC50).

### 2.5. Calculation of the IC50, ICr0, and ICrmed Indices

**Figure 2:**
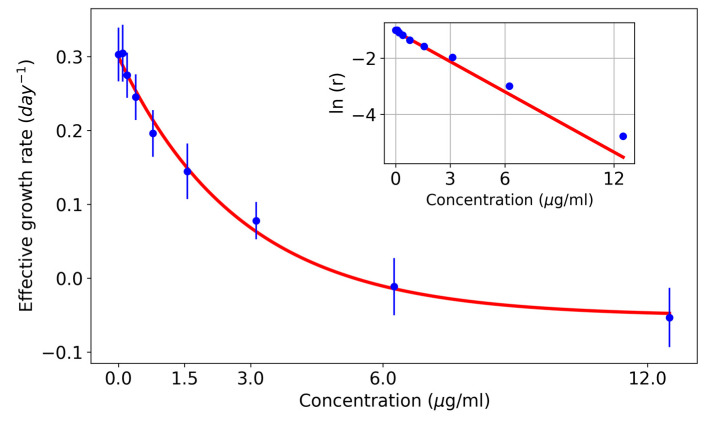
Concentration dependence of the effective growth rate in the HCT116 cell line. An exponential decrease is shown. The inset shows the same plot on a semilogarithmic scale. Note that in a semilogarithmic scale plot, data points can be fitted with a linear regression. Three independent experiments were performed, each including three technical replicates.

As can be seen in Figure 2, the plot of the growth rate vs. drug concentration shows a rapid initial decrease followed by a slower decrease for high concentrations. In most of the experiments (shown in a semi-logarithmic scale and shifted to avoid negative values), one can observe that the logarithm of the growth rate decreases approximately linearly with time, so it can be fitted by linear regression (see the inset in Figure 2). The exponential decline in cell populations treated with drugs has been also demonstrated in other studies [25]. When this fit is adequate, a mathematical model of the dependence of the growth rate on the drug concentration can be obtained. With this model, the concentration for a given growth rate can be calculated as follows:

(6) $$r(C)+r_{\infty }=A\cdot e^{-\alpha \cdot C},$$

where $r_{\infty }$ is the negative shifted value (at very high drug concentrations, the growth rate is negative) and α is the exponent. By inverting Equation (6), the drug concentration for a given effective growth rate can be determined as follows:

(7) $$C(r)=\frac{-1}{\alpha }ln\left(\frac{r-r_{\infty }}{A}\right),$$

Let us maintain that we have calculated an effective growth rate for different drug concentrations using the bootstrapping technique. The bootstrapping technique provides a distribution of the effective growth rate from which we can obtain its mean and confidence intervals. Now, we can also use the bootstrapping technique in the exponential fit of growth rates across different drug concentrations with this empirical distribution. In this instance, we have performed a mixed bootstrap regression model. We have fixed the regressor values (as in the residual bootstrap method), and the response variable, $r_{i}^{*}$, is generated by Monte Carlo simulation from the slope distribution as a parametric bootstrap (obtained in the previous effective growth rate calculation). From each bootstrapping regression, we obtain the fitting parameters (A, *α*, $r_{\infty }$) needed in Equation (7) to obtain the corresponding concentration for the IC50 and other indices. With the $N_{b}$ bootstrap simulations, an index distribution can be obtained, from which the expected value and the confidence intervals can be calculated. Monte Carlo simulations were carried out with the same libraries as described in the bootstrap method.

In the Mathematical Model section, we have found an expression for the effective growth rate corresponding to 50% viability, $r_{IC50}$ (Equation (6)). The IC50 index is defined as the drug concentration that will produce 50% cell viability, so Equation (7) can be used to calculate IC50 (substituting r by $r_{IC50}$). Similarly, ICr0 and ICrmed can be calculated by substituting in Equation (7) r by 0 and $r_{C}/2$, respectively.

In summary, in order to obtain the IC50 index (and the other new indices), the following steps are required: (i) determine the corresponding effective growth rate for that index; (ii) from the growth rate distribution, obtain a sample of r for each drug concentration and perform a shifted exponential fit; (iii) using Equation (7), calculate the concentration for $C(r_{IC50})$; and (iv) repeat steps (ii) and (iii) a large number of times (we recommend 1000–5000 times) to obtain a distribution of concentrations for $r_{IC50}$. From this distribution, we can obtain the mean and confidence intervals.

To validate the IC50 index value obtained with this new procedure, it was compared to the IC50 parameter as calculated by two conventional methods: GraphPad Prism 8.0 (GraphPad Software Inc., San Diego, CA, USA) and the IC50 calculator web program (https://www.aatbio.com/tools/ic50-calculator, accessed on 12 January 2025). Both tools perform data fitting by assuming that the response curve follows a symmetrical sigmoidal shape using a four-parameter logistic regression model (this value is also referred to as the relative IC50 parameter) as follows:

(8) $$Y=Y_{min}+\frac{Y_{max}-Y_{min}}{1+{\left(\frac{X}{IC50}\right)}^{H}},$$

### 2.6. Statistical Analysis

Wilcoxon tests were used to compare the statistical differences between two paired groups. A *p*-value < 0.05 was employed in all tests. GraphPad Prism 8 (GraphPad Software Inc., San Diego, CA, USA) was employed to perform the statistics and process the images.

## 3. Results

### 3.1. Viability and IC50 Calculation Are Time and Method Dependent

**Figure 3:**
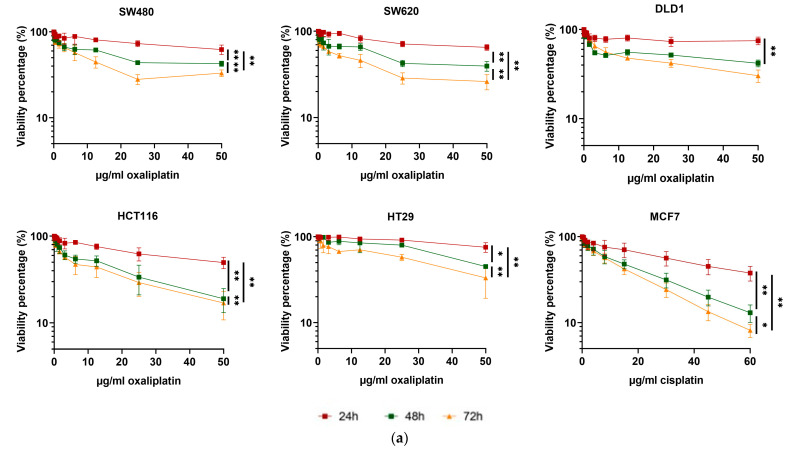

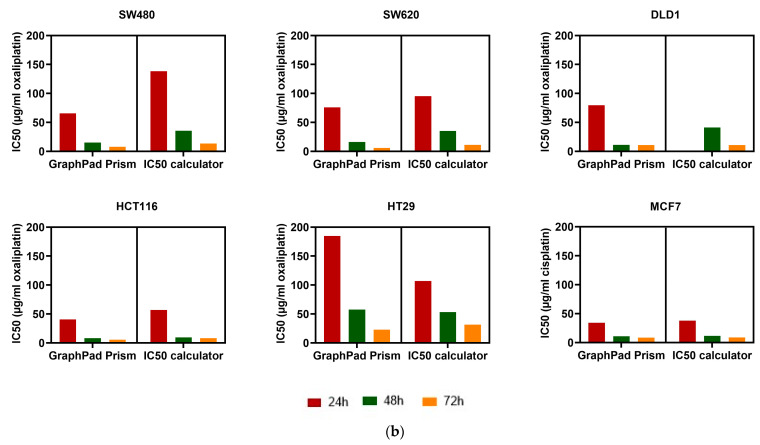
Response to chemotherapy is time dependent in different cell lines. (**a**) Viability was quantified in six cancer cell lines at three different endpoints (24, 48, and 72 h). Differences were evaluated by the Wilcoxon test. The probability is indicated by * *p* < 0.05, and ** *p* < 0.01. (**b**) IC50 value was calculated by GraphPad Prism and IC50 calculator programs in six cancer cell lines for three endpoints (24, 48, and 72 h). Three independent experiments were performed, each including three technical replicates.

The percentage of viable cells was measured at three time points (24, 48, and 72 h) when exposed to a wide range of chemotherapy doses (oxaliplatin 0–50 µg/mL or cisplatin 0–60 µg/mL). Six cancer cell lines were employed: SW480, SW620, DLD1, HCT116, and HT29 from colorectal cancer and MCF7 from breast cancer. A decrease in viability in a dose-dependent manner, but also depending on the endpoint time, is shown in Figure 3a. Significant differences were found when comparing 24, 48, and 72 h endpoints in every cell line. The IC50 was calculated using both GraphPad and IC50 calculator programs. The results plotted in Figure 3b present differences between the 24, 48, and 72 h endpoints both by GraphPad Prism and IC50 calculator programs (significant differences were not evaluated, as the calculator provides a value without error).

### 3.2. Effective Growth Rate Calculation

**Figure 4:**
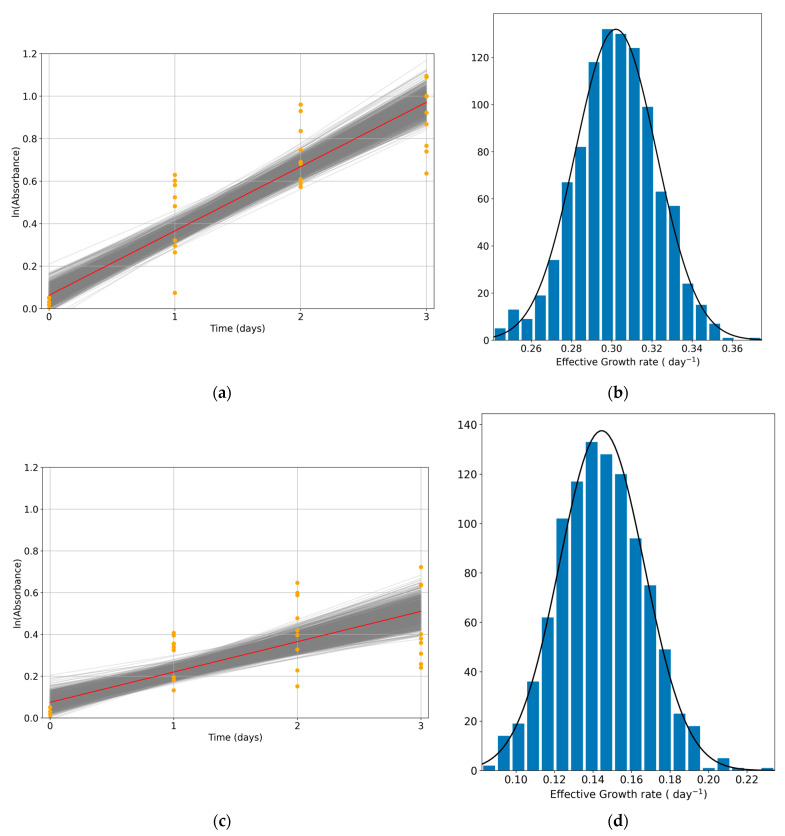
Growth rate calculation and distribution in the HCT116 colorectal cancer cell line. (**a**) Growth rate calculation for HCT116 control condition. A total of 1000 linear regressions were performed over the paired bootstrap samples of the experimental data (shown as orange dots). Each gray line corresponds to each bootstrap linear regression, and the red line represents the mean of these fits. (**b**) Distribution of the bootstrap linear regression slopes in HCT116 control data. (**c**) Growth rate calculation for HCT116 with oxaliplatin at a 1.56 µg/mL concentration. (**d**) Distribution of the bootstrap linear regression slopes in HCT116 with oxaliplatin at a 1.56 µg/mL concentration. Three independent experiments were performed, each including three technical replicates.

To determine whether a cell colony is proliferating or declining, the population evolution was assessed. Here, we calculated the effective growth rate both in the control (without treatment) and in a range of drug concentrations. First, the exponential growth condition had to be verified. Control data are plotted on a semi-logarithmic scale to verify this exponential trend. The HCT116 cell line was used in Figure 4a for this purpose; in this figure, we see exponential growth in HCT116, as it depicts a linear trend when plotting on the semilog scale. This result ensures that external factors were not affecting the experiment, and the effect was due to the variable of interest. The experimental data are shown as orange dots, and the fit for these data is shown as a red line. For each experimental condition, 1000 bootstrap linear regressions were performed to obtain the growth rate distribution (each gray line corresponds to the linear fit of one bootstrap sample). The distribution of the bootstrap linear regression slopes is shown in Figure 4b, presenting a normal distribution of the data. Similar results were obtained at different drug concentrations (as an example, a concentration of 1.56 µg/mL of oxaliplatin is shown in Figure 4c,d). 

### 3.3. Effective Growth Rate Decreases in an Exponential Dose-Dependent Manner

The effective growth rate for the HCT116 cell line under different oxaliplatin concentrations is shown in Table 1. The growth rate turns from positive to negative as the oxaliplatin concentration gets higher. A control growth can be assigned to a cell line, and the pattern of the change in the growth rate could define the response to a treatment.

**Figure 5:**
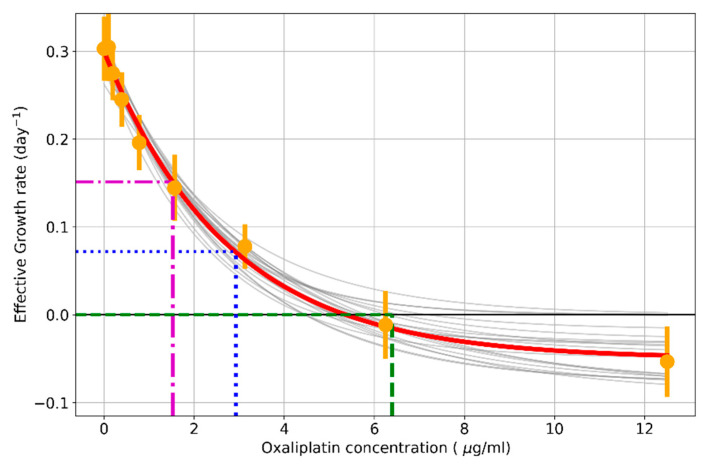
The effective growth rate decreases in an exponential dose-dependent manner in the HCT116 cell line. A total of 1000 Monte Carlo exponential regressions were performed from the slope distribution obtained in the previous effective growth rate calculation (shown as orange dots). Each gray line corresponds to each bootstrap exponential regression, and the red line is the mean of these fits. The parameters to measure resistance are shown in dashed lines: IC50 (blue), ICr0 (green), and ICrmed (purple).

An exponential dose-dependent decrease in the growth rate was found in all the studied cell lines. Then, 1000 Monte Carlo exponential regressions from the slope distribution obtained in the previous effective growth rate calculation were simulated. As an example, the exponential fit for the HCT116 cell line is shown in Figure 5, where each gray line is an exponential regression fit. The results for the rest of the cell lines are shown in Appendix A.1. These data will be employed to calculate indices that may be useful to compare the response to treatment between cell lines or different culture conditions.

### 3.4. Parameters to Measure Resistance to Drugs

IC50 value is a conventional parameter that corresponds to the dose at which cell viability is 50%, i.e., the sample population is 50% of the control population. Its calculation was performed by three different methods: GraphPad Prism, IC50 calculator, and the new method proposed in this article, which uses the cell growth rate. It was calculated for the six cell lines employed, and the results are shown in Table 2. Both conventional methods provided values that differ from each other as well as from the value proposed in this article. This parameter is both time and method dependent.

**Figure 6:**
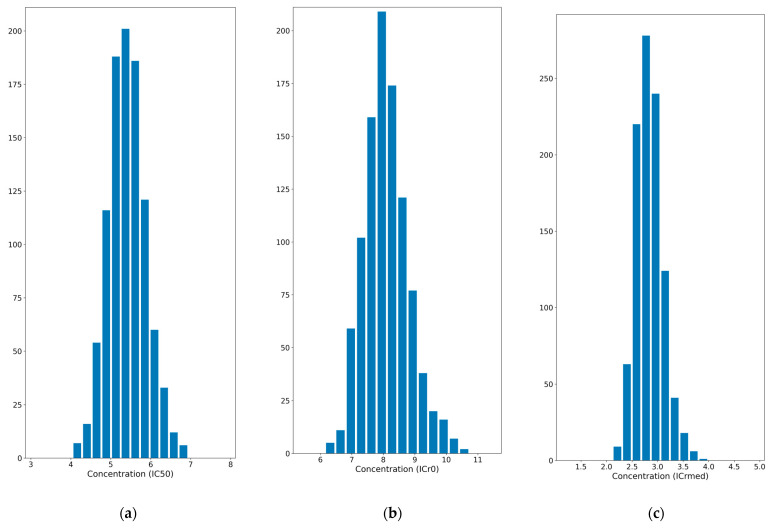
Distribution of the parameters to measure cell lines’ resistance to drugs. IC50 (**a**) ICrmed (**b**) and ICr0 (**c**) distributions are shown for the HCT116 cell line.

Moreover, we propose two new parameters to study drug resistance. The ICr0 value is the dose at which the population is neither growing nor dying, so the growth rate is 0 (called r0). For its part, the ICrmed value corresponds to the dose at which the population is growing at half the rate of the control (called rmed). These parameters are not time dependent by definition, as they are calculated from the growth rate at different drug concentrations. IC50, ICr0, and ICrmed values were obtained for each Monte Carlo exponential regression, resulting in the distribution shown in Figure 6 for the HCT116 cell line and in Appendix A.2 for the rest of the cell lines. The means of these values and the 90% confidence intervals are shown in Table 3. ICr0 and ICrmed classify the cell lines in terms of resistance with the same pattern as conventional IC50. They are similar parameters, but the use of ICrmed is more recommended for highly resistant cell lines, as they may never reach r0 in the experimental timeframe (if they continue growing at the studied doses). The higher the value, the more resistant the cell line is. This allows comparison between cell lines or culture conditions, depending on the experiment.

## 4. Discussion

The continuing need to identify treatments for highly incident cancers, as well as their process of optimization, has led to the development of multiple methods of analysis of potential treatments. To reduce the risk when moving to clinical trials, initial steps require an in vitro study of the compounds’ effects in analogous systems, such as on human-derived immortalized cell lines, and their subsequent use in in vivo assays [26]. Pharmacological development focuses on the effects of a compound on the pathology target, and these effects are measured by cell proliferation, apoptosis, or metabolic activity [27,28]. Amongst these typically studied effects, we can highlight cell viability. This is an important measure, particularly in cancer, where a treatment’s efficacy relies on its capacity to reduce the tumor size [29,30]. Consequently, ideal treatment conditions will decrease cancer cell viability without affecting healthy cell viability. Since pharmacodynamics seeks to understand cellular behavior over a given period, models focused on an endpoint response will only serve to compare compounds. Due to these limitations, such models can lead to imprecise interpretations [31]. Therefore, a model that incorporates data on temporal and dose-dependent changes in cell growth allows for a better understanding of treatment efficacy, especially in cancers that develop resistance.

The most widely used index to define the effects of a compound on cell viability is the value of the half-maximal inhibitory concentration (IC50). This value is an estimator of the potency of a substance and is defined as the concentration at which a biological process is inhibited by 50% [32]. Thus, when used as a measure of cell viability, IC50 indicates how much of the compound is required to reduce the number of viable cells by half. In this context, the index has been used to i) estimate treatment efficacy, ii) confirm a lack of detrimental effects, and iii) compare the behavior of different cell lines when exposed to the same compound [33,34,35]. For example, the IC50 has traditionally served as a measure of resistance to anti-cancer drugs [36]. An important advantage of the index is its easy calculation, simply determined as the concentration at which the ratio between the number of viable treated cells and the number of viable control (untreated) cells is 50%. However, the IC50 as a measure of anti-cancer efficacy has been shown to have multiple weaknesses [37,38]. Among them, we highlight the time-dependent nature of this index, where variations of relatively few hours to the endpoint of an assay will result in a different IC50 value, as shown in Table 1 and previously described in the literature [39,40,41,42]. This is a result of the comparison of two populations (sample and control), which normally evolve over time with different growth rates [43,44]. When reproducing dose–response assays, different values are obtained between studies. In this sense, the IC50 value does not accurately reflect the temporal dynamics of the cellular response; in fact, it inherently assumes a homogeneous response by only evaluating the endpoint [29].

Our findings show, in six different cancer cell lines (Figure 3b), that there are significant differences in the calculated IC50 value, depending on the endpoint at which the percentage of viable cells has been calculated (24, 48, or 72 h). In addition, when comparing three different methods of IC50 calculation (GraphPad Prism [45], IC50 calculator [46], and our mathematical model), the results for the same time point and cell line also vary. It is worth noting that our model’s IC50 values are typically lower than those calculated in endpoint models. This pattern has been previously observed by Hazekawa et al. [41], who use a continuous monitoring system to obtain real-time values of cell viability, consequently obtaining lower IC50 values than those they had estimated in endpoint assays. This real-time monitoring is analogous to our effective growth rate estimation, as it decreases the influence of growth rate-altering culture conditions.

The biological meaning of the IC50 index is also difficult to interpret, as it cannot distinguish between populations that will continue to grow or those in which the cells die. According to the study by Özkaya et al., there may be two causes for the decrease in cell viability results: the rate of cell division and cell death. To address this issue, our method includes determining the change in cell proliferation among viable cells [47]. In our mathematical model (where $r_{c}$ corresponds to the growth rate of the chemotherapeutic-treated cells), if we assume an equal initial population between control $\left(N_{0}^{*}\right)$ and treated cells ($N_{0C}$) and look at the growth rate of our cells when there is 50% viability (V = 0.5) after 3 days (t = 3) (the time at which the IC50 index is typically calculated), then

(9) $$(\mathrm{Model})\;r_{C}\left(V,t\right)=r_{0}+\frac{1}{t}\cdot ln\left(V\right),$$

(10) $$(50\%\;\mathrm{viability},\;3\;\mathrm{days})\;r_{C}\left(V=0.5,t=3\right)=r_{IC50}=r_{0}-0.231$$

The model thus proves that a viability value of 50% can be obtained with high or low cell proliferation. That is to say, the growth rate can be positive (and the colony population continues to grow) or negative (colony cells die and the population decreases), depending on the untreated cells’ growth rate, $r_{0}$.

To this end, we have endeavored to find biologically meaningful parameters of cell proliferation to indicate the effects of chemotherapeutic compounds in a time-independent manner. Cells’ exponential growth can be represented as a differential equation where, rather than defining the size of a population at a given time, it is indicated as the rate at which the population increases in a particular amount of time (the growth rate) [43]. When considering short time frames, i.e., when growth is not affected by space, nutrients, or any other constraints, this rate (the effective growth rate) is constant, although considerations, such as density dependence, mean that this value is altered across longer time frames [44]. The proposed mathematical model evaluates whether the control exhibits exponential growth, assuring that no external factors are influencing the culture. When referring to exponential growth, we consider the overall population rather than individual cells, as some cells will reproduce while others may not. Our model does not rely on synchronized cell growth but instead determines whether the overall population grows in an exponential manner. The model evaluates whether this condition is met and generates an error if the data fail to fit an exponential curve. This functionality is not available in widely used IC50 calculators, highlighting a limitation of the existing methods and a key advantage of our approach. Rather than producing unreliable results, our method provides an error message, thereby ensuring data integrity.

After confirming the condition of exponential growth and Gaussian distribution of the data, the proposed model determines the effective growth rate of cells when exposed to a range of drug concentrations in a time-independent manner. The dose-dependent reduction in growth rates in this model, as observed in all six of our cell lines, thus allows for a more robust method of study of in vitro response to drugs, as the use of bootstrapping methods means that values are the result of thousands of iterations of the fit to the curve. This larger number of iterations helps to reduce the error of the fitting parameters.

It is worth noting that the application of our model for different compounds does require slightly more time upfront than conventional IC50 calculations. The procedure is the same as the conventional method and the results will still be available within the same overall timeframe as the previous method, but it requires more data inputs at different timeframes, slightly increasing bench time (Appendix A.3). We consider this a fair trade off for the robustness of the acquired value, as our model calculates an estimated error, unlike the IC50 calculator, and this error is much lower than the error obtained for the same conditions using the GraphPad Prism program (see Table 2). This decreased error in conventional IC50 calculation allows for more precise comparison between cell lines and increased reproducibility of assays. When relying on a single endpoint (as in the conventional method), the results are often inconsistent, unreliable, and prone to significant errors. If any issues occur, the entire experiment will need to be discarded, requiring a new attempt, which would ultimately be more costly and time consuming than the experimental design we propose. So, the results obtained with the new proposed method are far more reliable and robust, eliminating the need to repeat experiments. This makes our method more cost effective in the long term. In addition, if time or materials are limiting factors, we have also tested the mathematical model using only the initial values and 72 h endpoint (similar to conventional methods) and are still able to estimate an approximate IC50 value. In Table 4, we can see that this reduction in data inputs does not have a significant impact on the error, although IC50 values do slightly differ.

To remedy the limitations of the IC50 index and establish a time-independent standardized parameter for the study of drug potency, we propose two new indices: ICr0 and ICrmed, both derived from the effective growth rate. ICr0 is defined as the value that indicates the compound concentration at which there is an effective growth rate of zero or the dose at which the treated cell population is neither growing nor dying. Drug concentrations above this threshold will thus result in a reduction in the population size. For its part, the other parameter that we propose, ICrmed, is the value that corresponds to the drug concentration that reduces the control population’s growth rate by half or the dose at which the treated population grows at half the rate of the control untreated cells.

When used to evaluate drug resistance, ICr0 and ICrmed classify the tested cell lines with the same pattern as conventional IC50, showing that they are adequate substitutes for this parameter (Table 3). However, unlike the IC50, their calculation is neither time nor method dependent. Conventional calculation of IC50 depends on the interpretation of dose–response curves, where the growth of cell lines is considered in direct relation to the absorbance of a reagent (such as MTT) and where the effect of the drug is calculated on the basis of a simple ratio between treated and untreated cells. However, this means that the results are directly dependent on seeding cell concentration, where any irregularities in seeding or concentrations where excessive cell density leads to cell death can invalidate the results [40,48]. Established programs to calculate IC50 rely on fitting a sigmoid function, which is, therefore, more sensitive to errors. Errors in its calculation not only affect drug research and development but can also have an impact on the safety and efficacy of applied therapies. Traditional methods often fail to detect errors, resulting in the use of inaccurate data. Our method, based on the growth rate at each point of the assay, is not as reliant on seeding concentration, leading to higher reproducibility, and it uses an empirical exponential function, which is less susceptible to errors. Plus, a unique characteristic of our model is its inclusion of a growth curve evaluation, confirming a condition of exponential growth prior to parameter estimation. In this way, as this new method provides additional time points for analysis, we can account for the possibility of non-ideal culture conditions and remove those data points that could confound the results, thus avoiding the need to repeat experiments altogether.

It is worth noting that although similar, we recommend the use of the ICrmed value over the ICr0 value for classification, as the ICr0 can be difficult to define for highly resistant cell lines. The use of the ICrmed allows for the evaluation of resistant cell lines that might never reach IC50 or r0 values in the experimental timeframe and removes the need to extrapolate this value, as would be performed in conventional IC50 calculations. When using these parameters, cell lines with higher values are classified as more resistant. This allows for direct comparison between cell lines or culture conditions, depending on the desired experimental outcome.

We have used these parameters to evaluate differences in colorectal cancer cell lines’ resistance to oxaliplatin. Oxaliplatin is a commonly used chemotherapeutic for the treatment of colorectal cancer; an alkylating agent, it affects the growth of cancer cells by damaging the DNA. Multiple molecular mechanisms of resistance to oxaliplatin have been described in tumors, including through accumulation in surrounding healthy cells or activation of diverse signaling pathways [49,50]. In our evaluation, comparing human colorectal cancer cell lines’ resistance to oxaliplatin treatment, all our parameters highlight DLD1 as an oxaliplatin-resistant line. The SW480 cell line would be highly resistant per the IC50 calculator’s value but not per our method or the GraphPad Prism value. In addition, while SW620 has an IC50 of about half that of DLD1 per the GraphPad value and our model’s estimators, this relation is flipped in the IC50 calculator’s estimates. The DLD1 cell line proceeds from a human colon adenocarcinoma in Duke’s C stage; for their part, SW-480 and SW-620 derive from the primary tumor and metastatic carcinoma of the same patient, respectively [51]. We have previously characterized these lines’ growth when exposed to oxaliplatin treatment and have seen similar patterns as those indicated by our model and the GraphPad Prism IC50 values [25].

In summary, any investigation studying the efficacy of drugs on different cell types as a pre-clinical in vitro assay benefits from our method. The results are more reproducible, reliable, and time independent compared to established methods, such as IC50 estimation using a sigmoid curve, which is commonly employed by validated tools, like IC50 calculator or GraphPad. The IC50 index, as conventionally calculated, has been used in a variety of applications in the field of oncology, including the characterization of novel treatments, drug dosage calculations, and description of synergetic treatments, among others [52,53]. However, the lack of reproducibility and significant drop-off of viable treatments when moving to clinical trials emphasizes the need for new parameters, with biologically relevant interpretation and reduced error [54]. Although traditional methods for analyzing dose–response data, such as calculating IC50, are still widely used, their limitations are well known and, in the absence of true alternatives, studies have turned to complementary approaches that can provide additional information [55,56]. One such approach is the use of non-linear models, such as log-logistic, log-normal, and Weibull models, to fit dose–response data [57]. Some computational tools, such as R [56], or modeling approaches based on non-linear models have been used for dose–response analysis [58].

## 5. Conclusions

In conclusion, although the inhibitory concentration IC50 remains a widely used metric, researchers have explored several complementary approaches to improve the analysis and interpretation of dose–response data, pointing to kinetic assays as a plausible improvement, as we do in the present study. Our mathematical model allows for more precise comparison between cell lines, reduces the impact of negative culture conditions on the assay, and increases reproducibility by decreasing the error. In addition, the use of estimates based on effective growth rates (such as ICr0 and ICrmed), in contrast to endpoint assay-based parameters, reduces the values of drug concentrations needed for an equivalent effect. This more conservative value could, in turn, i) reduce the amount of the compound needed for treatment and reduce costs and ii) reduce the risk of harmful effects on surrounding healthy cells when testing compounds in vivo.

## Figures and Tables

**Table 1:** Effective growth rate and the confidence intervals at 90% for the HCT116 cell line under different oxaliplatin concentrations.

Concentration (µg/mL)	Effective Growth Rate (Days^−1^)	Standard Error
0	0.30	0.04
0.1	0.30	0.04
0.2	0.27	0.03
0.4	0.25	0.03
0.8	0.20	0.03
1.6	0.14	0.04
3.13	0.08	0.03
6.25	−0.01	0.04
12.5	−0.05	0.04
25	−0.21	0.06
50	−0.44	0.04

**Table 2:** Parameters to measure cell lines’ resistance to drugs. IC50 was calculated by three methods: GraphPad Prism, IC50 calculator, and the new method described in this article.

Cell Line	IC50 GraphPad Prism (µg/mL)	IC50 Calculator (µg/mL)	IC50 New Method (µg/mL)
SW480	7.89 [5.61–11.11]	13.52	5.8 ± 1.3
SW620	5.83 [3.90–8.71]	11.26	5.5 ± 2.1
DLD1	10.85 [8.57–13.76]	10.92	7.8 ± 2.2
HCT116	5.37 [3.95–7.29]	8.22	5.5 ± 1.0
HT29	22.88 [15.90–33.31]	31.54	25.5 ± 4.7
MCF7	8.44 [7.21–9.87]	8.97	11.1 ± 1.1

**Table 3:** Parameters to measure cell lines’ resistance to drugs. IC50, ICr0, and ICrmed were calculated by the new method described in this article. Means and 90% confidence intervals are shown.

Cell Line	IC50 (µg/mL)	ICr0 (µg/mL)	ICrmed (µg/mL)
SW480	5.8 ± 1.3	8.9 ± 2.0	2.7 ± 0.6
SW620	5.5 ± 2.1	6.3 ± 2.4	1.8 ± 0.7
DLD1	7.8 ± 2.2	16.0 ± 5.0	4.3 ± 1.2
HCT116	5.5 ± 1.0	8.3 ± 1.5	2.9 ± 0.5
HT29	25.5 ± 4.7	37.0 ± 10.0	12.0 ± 3.0
MCF7	11.1 ± 1.1	13.3 ± 1.3	5.2 ± 0.9

**Table 4:** Parameters to measure cell lines’ resistance to chemotherapeutics for different endpoints.

Cell Line	Endpoints	IC50 (µg/mL)	ICr0 (µg/mL)	ICrmed (µg/mL)
SW480	0 h, 24 h, 48 h, and 72 h	5.8 ± 1.3	8.9 ± 2.0	2.7 ± 0.6
	0 h and 72 h	5.9 ± 1.4	8.7 ± 2.1	2.7 ± 0.6
SW620	0 h, 24 h, 48 h, and 72 h	5.5 ± 2.1	6.3 ± 2.4	1.8 ± 0.7
	0 h and 72 h	6.0 ± 1.8	6.7 ± 2.0	1.9 ± 0.6
DLD1	0 h, 24 h, 48 h, and 72 h	7.8 ± 2.2	16.0 ± 5.0	4.3 ± 1.2
	0 h and 72 h	7.7 ± 2.2	15.8 ± 4.3	4.3 ± 1.2
HCT116	0 h, 24 h, 48 h, and 72 h	5.5 ± 1.0	8.3 ± 1.5	2.9 ± 0.5
	0 h and 72 h	5.6 ± 0.8	8.3 ± 1.2	2.9 ± 0.4
HT29	0 h, 24 h, 48 h, and 72 h	25.5 ± 4.7	37.0 ± 10.0	12.0 ± 3.0
	0 h and 72 h	23.7 ± 4.4	34.4 ± 10.3	10.6 ± 2.9
MCF7	0 h, 24 h, 48 h, and 72 h	11.1 ± 1.1	13.3 ± 1.3	5.2 ± 0.9
	0 h and 72 h	11.5 ± 1.1	12.9 ± 1.1	5.0 ± 0.8

**Figure A1:**
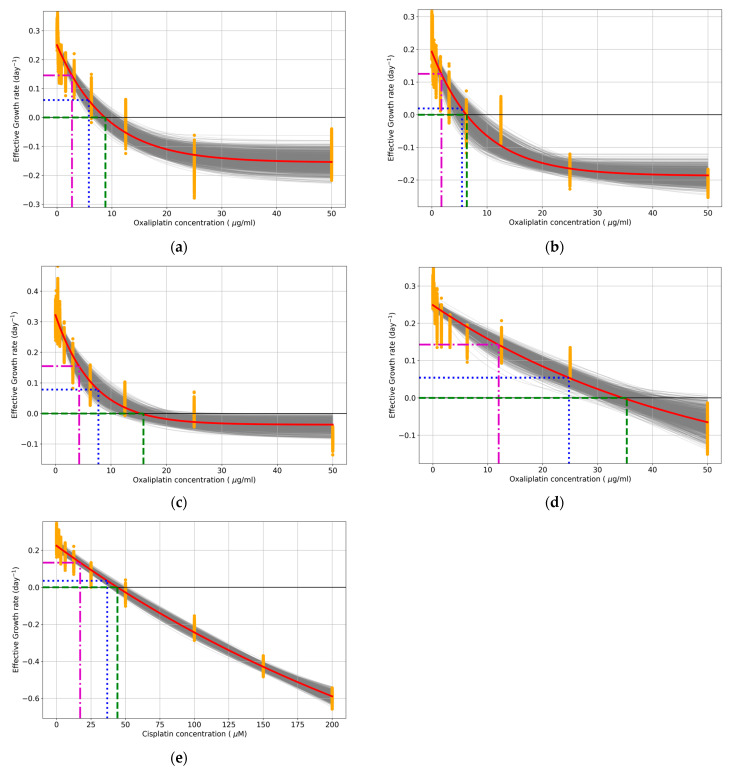
The effective growth rate decreases in an exponential dose-dependent manner. The experiment was performed in five cancer cell lines: (**a**) SW480, (**b**) SW620, (**c**) DLD1, (**d**) HT29, (**e**) MCF7. A total of 1000 Monte Carlo exponential regressions were performed from the slope distribution obtained in the previous effective growth rate calculation (shown as orange dots). Each gray line corresponds to each bootstrap exponential regression, and the red line is the mean of these fits. The parameters to measure resistance are shown in dashed lines: IC50 (blue), ICr0 (green), and ICrmed (purple).

**Figure A2:**
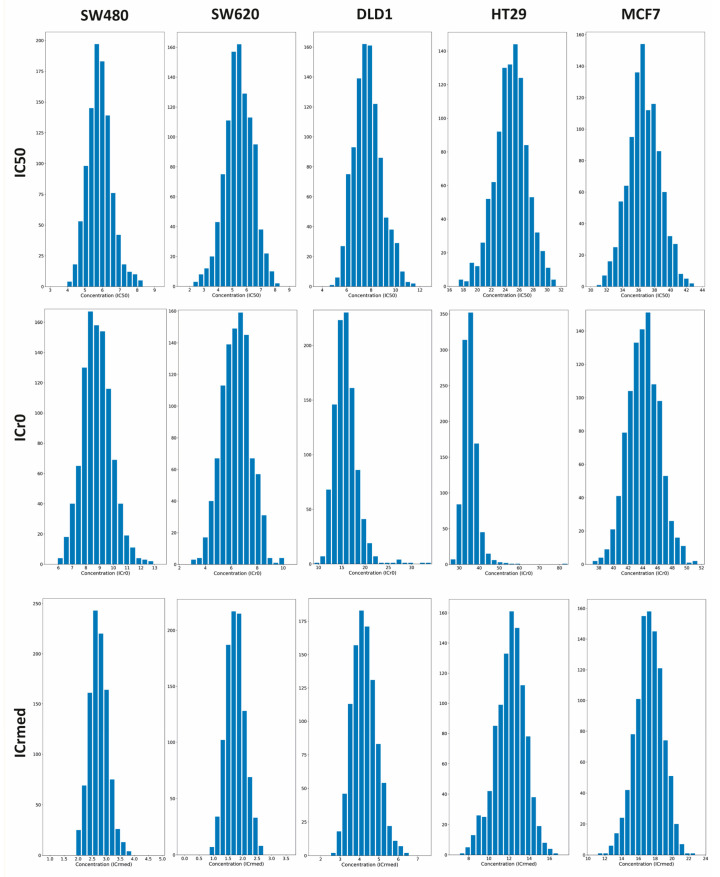
Distribution of the parameters to measure cell lines’ resistance to drugs. IC50, ICrmed, and ICr0 distributions are shown for SW480, SW620, DLD1, HT29, and MCF7 cancer cell lines.

**Figure A3:**
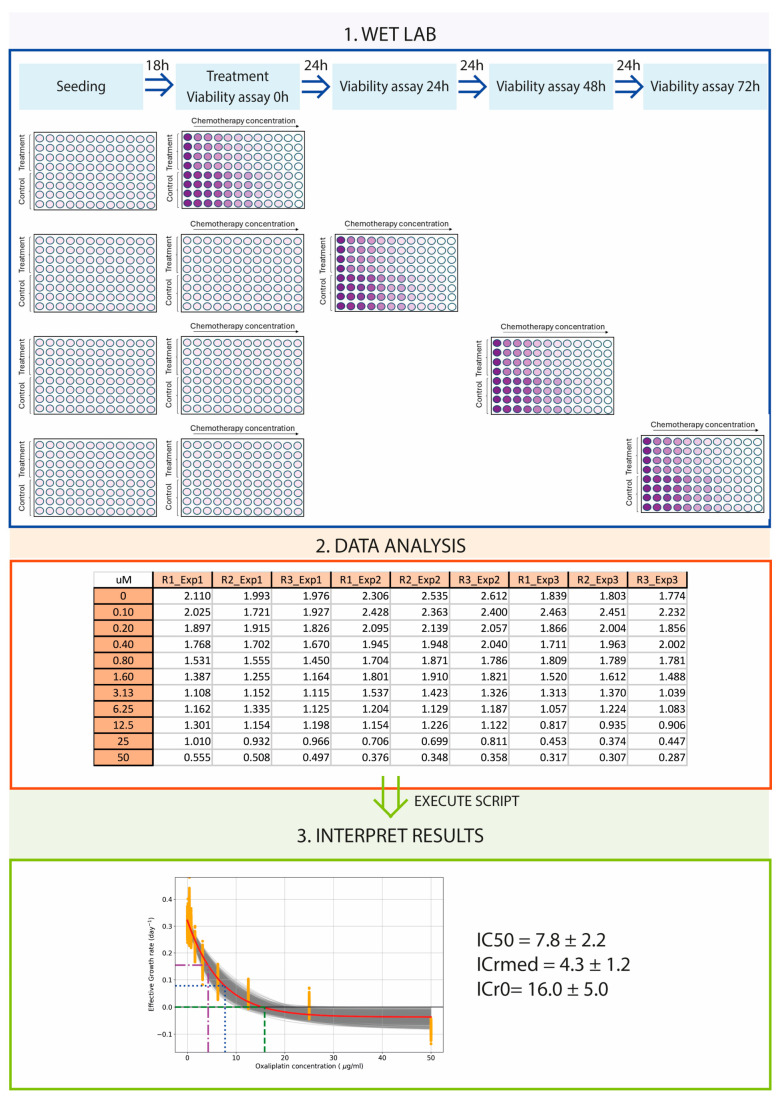
Diagram of the procedure for the new proposed method.

## Data Availability

Supporting data are available upon direct request to the authors. The Python scripts are available from GitHub (https://github.com/JJ-Lab/CSC_IC50).
